# Prevalence of Arthritis in Africa: A Systematic Review and Meta-Analysis

**DOI:** 10.1371/journal.pone.0133858

**Published:** 2015-08-04

**Authors:** Anthony Usenbo, Veronika Kramer, Taryn Young, Alfred Musekiwa

**Affiliations:** 1 Community Health Division, Faculty of Medicine and Health Sciences, Stellenbosch University, Cape Town, South Africa; 2 Cochrane Infectious Diseases Group, Liverpool School of Tropical Medicine, Liverpool, United Kingdom; 3 Centre for Evidence Based Health Care, Faculty of Medicine and Health Sciences, Stellenbosch University, Cape Town, South Africa; VU University Medical Center, NETHERLANDS

## Abstract

**Objective:**

In this systematic review, we estimate the prevalence of six types of arthritis in Africa; namely rheumatoid arthritis, osteoarthritis, juvenile arthritis, psoriatic arthritis, gout, and ankylosing spondylitis.

**Methods:**

We comprehensively searched literature on 31 August 2014 in MEDLINE, EMBASE, Web of Science and the Cochrane Library to identify eligible studies from 1975 up to 31 July 2014. Two review authors independently selected studies, extracted data, and appraised studies. We carried out random effects meta-analysis of prevalence of arthritis and assessed heterogeneity through subgroup analyses. We performed separate analyses for population- and hospital-based studies, as well as rural and urban settings.

**Main Findings:**

We included 27 cross-sectional studies (20 population-based and 7 hospital-based) from Africa reporting on the prevalence of arthritis. The majority of the studies were from South Africa (44.4%, 12/27). Rheumatoid arthritis in urban settings ranged from 0.1% in Algeria, 0.6% in the DRC, to a meta-analysis overall prevalence of 2.5% in South Africa, and in rural settings ranged from a meta-analysis overall prevalence of 0.07% in South Africa, 0.3% in Egypt, to 0.4% in Lesotho. Osteoarthritis was the most prevalent form of arthritis and in urban settings it was 55.1% in South Africa and in rural settings, all in South Africa, ranged from 29.5%, 29.7%, up to 82.7% among adults aged over 65 years. Other results include highest prevalence of 33.1% for knee osteoarthritis in rural South Africa, 0.1% for ankylosing spondylitis in rural South Africa, 4.4% for psoriatic arthritis in urban South Africa, 0.7% for gout in urban South Africa, and 0.3% for juvenile idiopathic arthritis in urban Egypt. A third of the included studies had a low risk of bias (33.3%, 9/27), 40.8% (11/27) moderate risk, and 25.9% (7/27) had a high risk of bias.

**Conclusions:**

In this systematic review, we have identified the paucity of latest prevalence data on arthritis in Africa. More studies are needed to address the prevalence and the true burden of this disease in Africa.

## Introduction

Arthritis is an inflammatory disorder affecting one or more joints of the body with varying causal factors, including trauma, infections, autoimmune disorders, idiopathic causes, and aging. Irrespective of the cause, the underlying pathophysiology involves the breakdown of cartilage, which protects the end surfaces of bones at the joints, leading to the loss of smooth glide at the joint during movement. This frictional rubbing results in pain, swelling and stiffness at the joint and eventual muscle strain due to difficulty moving the joint [1].

There are six main types of arthritis. Rheumatoid arthritis is a systemic autoimmune disease that presents as a symmetrical inflammatory polyarthritis which affects the smaller joints such as hands and feet first, before affecting larger joints [2]. Osteoarthritis is characterized by degeneration of the joints such as the knee and the hip [3]. Juvenile arthritis comprises a range of arthritic disorders affecting children and adolescents below the age of 16 years [4].Psoriatic arthritis is a form of arthritis affecting people with psoriasis, a skin disorder [5]. Gouty arthritis, or simply gout, is associated with the deposition of monosodium urate crystals in the tissues and joints [6]. Ankylosing spondylitis is an axial arthritis, which affects the vertebra causing inflammatory spinal pain and limited spinal and chest wall movements [7].

Prevalence data on arthritis in Africa remain scarce. Available studies reporting on prevalence have a wide range of estimates partly due to methodological differences and geographic or regional variation [8, 9]. Disability due to musculoskeletal disorders has increased by 45% from 1990 to 2010 and osteoarthritis is listed as the fastest increasing major health condition and ranked second as cause of disability by World Health Organization (WHO) [10].

To estimate the burden of disability caused by arthritis, an accurate population prevalence record is required [11]. Despite the overwhelming reports on the rising prevalence of musculoskeletal conditions, data from Africa are lacking and underestimated. In estimating the burden of rheumatoid arthritis in Africa in 2006 small studies from Nigeria, Liberia, and South Africa were used, which showed a high male to female ratio that was inconsistent with global trends and literature [2]. Similarly in estimating the burden for osteoarthritis in Africa only one study from South Africa was used, emphasizing the paucity of data in Africa [12].

## Objective

The objective of this systematic review was to determine, from available literature sources, the prevalence of arthritis in Africa.

## Methods

### Eligibility criteria

In this review, we considered cross sectional surveys carried out in Africa reporting on the prevalence of arthritis in all age groups. We included both population-based and hospital-based studies. We excluded studies that were not cross-sectional, had unclear denominators, did not deal with any of the six types of arthritis listed, or were published before 1975 and therefore considered less reflective of up-to-date disease frequency and distribution.

### Identifying studies for this review

An information specialist conducted electronic searches on 31 August 2014 in MEDLINE, EMBASE (OVID), Web Of Science and The Cochrane Library for studies published from January 1975 to 31 July 2014 using the following subject-specific search terms: “arthritis”[MESH] AND arthritis/or rheumatoid arthritis/or arthritis.mp. or psoriatic arthritis/ or juvenile rheumatoid arthritis/ or osteoarthritis.mp or osteoarthritis/ or ankylosing spondylitis.mp. or ankylosing spondylitis/ or gout.mp. or gout”. Geographic filters for African countries were applied [13]. The full strategy, which was run in Endnote software, and the terms used are reported in Table 1. The lead author searched for studies available only in print form at the Walter Sisulu University and Stellenbosch University libraries in South Africa with the assistance of experienced librarians. We also screened reference lists of included studies for additional eligible studies. We did not apply any language restrictions.

**Table 1:** Search strategy for arthritis prevalence studies from African countries.

1	arthritis/ or rheumatoid arthritis/ or arthritis.mp. or psoriatic arthritis/ or juvenile rheumatoid arthritis/
2	osteoarthritis.mp. or osteoarthritis/
3	ankylosing spondylitis.mp. or ankylosing spondylitis/
4	gout.mp. or gout/
5	lupus erythematosus/
6	1 or 2 or 3 or 4 or 5
7	risk factors.mp. or risk factor/
8	prevalence/
9	7 or 8
10	6 and 9
11	"Africa south of the Sahara"/ or Central Africa/ or Africa/ or South Africa/ or North Africa/
12	(somalia or south africa or "sthelena" or sudan or swaziland or tanzania or togo or tunisia or uganda or "western sahara" or zaire or zambia or zimbabwe or "Sierra Leone").mp.
13	(algeria or angola or benin or botswana or burkinafaso or burundi or cameroon or canary islands or cape verde or central african republic or chad or comoros or congo).mp.
14	(djibouti or egypt or equatorial guinea or eritrea or ethiopia or gabon or gambia or ghana or guinea or guinea bissau or ivory coast or cote d ivoire or jamahiriyaor jamahiriya or kenya or lesotho or liberia or libya or libya or madagascar or malawi or mali or mauritania or mauritius or mayotte or morocco or mozambique).mp.
15	(namibia or niger or nigeria or principe or reunion or rwanda or sao tome or senegal or seychelles).mp.
16	11 or 12 or 13 or 14 or 15
17	10 and 16

### Study selection, data extraction, and quality assessment

Two review authors independently screened the search output and retrieved full texts of potentially relevant studies and assessed eligibility with a pre-piloted eligibility form. We resolved disagreements by discussion.

We independently extracted prevalence percentage with 95% confidence interval, type of arthritis, age, gender ratio, start and end dates of study, country, study design, setting and study population of each included study using a pre-piloted data extraction form. We resolved disagreements by verification and discussion. We assessed the risk of bias of each study using the Hoy 2012 tool with ten parameters addressing internal and external validity (Table 2)[14]. Each parameter was assessed as either low or high risk of bias. Unclear was regarded as high risk of bias. The overall risk of bias was then scored according to the number of high risk of bias parameters per study: low (≤2), moderate (3–4), and high (≥ 5).

**Table 2:** Risk of bias assessment tool.

1. Representation	
Was the study population a close representation of the national population?	Yes (low risk)
	No (high risk)
	Unclear
2. Sampling	
Was the sampling frame a true or close representation of the target population?	Yes (low risk)
	No (high risk)
	Unclear
3. Random selection	
Was some form of random selection used to select the sample OR was a census undertaken?	Yes (low risk)
	No (high risk)
	Unclear
4. Non response bias	
Was the likelihood of non-response bias minimal?	Yes (low risk)
	No (high risk)
	Unclear
5. Data collection	
Were data collected directly from the subjects?	Yes (low risk)
	No (high risk)
	Unclear
6. Case definition	
Was an acceptable case definition used in the study?	Yes (low risk)
* *	No (high risk)
	Unclear
7. Reliability and validity of study tool	
Was the study instrument that measured the parameter of interest show to have reliability and validity?	Yes (low risk)
	No (high risk)
	Unclear
8. Data collection	
Was the same mode of data collection used for all subjects?	Yes (low risk)
	No (high risk)
	Unclear
9. Prevalence period	
Was the length of the prevalence period for the parameter of interest appropriate?	Yes (low risk)
	No (high risk)
	Unclear
10. Numerators and denominators	
Were the numerator(s) and denominator(s) for the parameter of interest appropriate?	Yes (low risk)
	No (high risk)
	Unclear

### Data synthesis and management

We analyzed population-based and hospital-based studies separately. Within each group of these two types of studies, we reported results separately for each type of arthritis and also for rural/urban settings. All the included studies focused on point prevalence. We calculated prevalence by dividing the number of observed cases of arthritis by the total number of observed respondents, and expressed as a percent. We used Microsoft Excel to calculate the 95% confidence interval for the prevalence, where these were not reported. We used the standard formula for calculating the standard error of a proportion [15], that is,

$$\sqrt{\frac{p\left(100-p\right)}{n}}$$

where *p* = prevalence (in %), and *n* = sample size. We assumed the distribution of the prevalence statistic to be Normal and used the critical value of 1.96 when calculating the 95% confidence intervals.

We performed random-effects meta-analysis since we expected variability in prevalence estimates from different studies. We assessed heterogeneity through the use of both the Chi-square test and the I-square test statistic. We considered a p-value of less than 0.10 to be significant for the Chi-square test due to the low power of this test and an I-square of at least 50% to be significant heterogeneity [16]. There were various case definitions from separate studies and we judged results to be clinically homogenous and we tested for statistical heterogeneity and only combined where heterogeneity was not statistically significant. We investigated sources of heterogeneity through subgroup analysis with respect to the country from which the study was done since prevalence of arthritis is known to have regional variation [17]. The small number of studies in the meta-analyses did not allow us to perform sensitivity analyses with respect to risk of bias, diagnostic criteria or age range. Only studies with similar risk of bias assessment were pooled in a meta-analysis and studies with high risk of bias assessment were excluded from meta-analysis. Where there was significant heterogeneity, meta-analysis was not performed. We performed all meta-analyses using STATA version 13 and displayed results in the form of forest plots. We used the STATA command ‘metan’ to perform meta-analysis.

There was no ethical clearance required for this study because all data have been published in journal articles. We registered and published the protocol for this review in PROSPERO, Registration number CRD 42013006035, available on the website http://www.crd.york.ac.uk/PROSPERO/

## Results

### Search for studies

**Fig 1:**
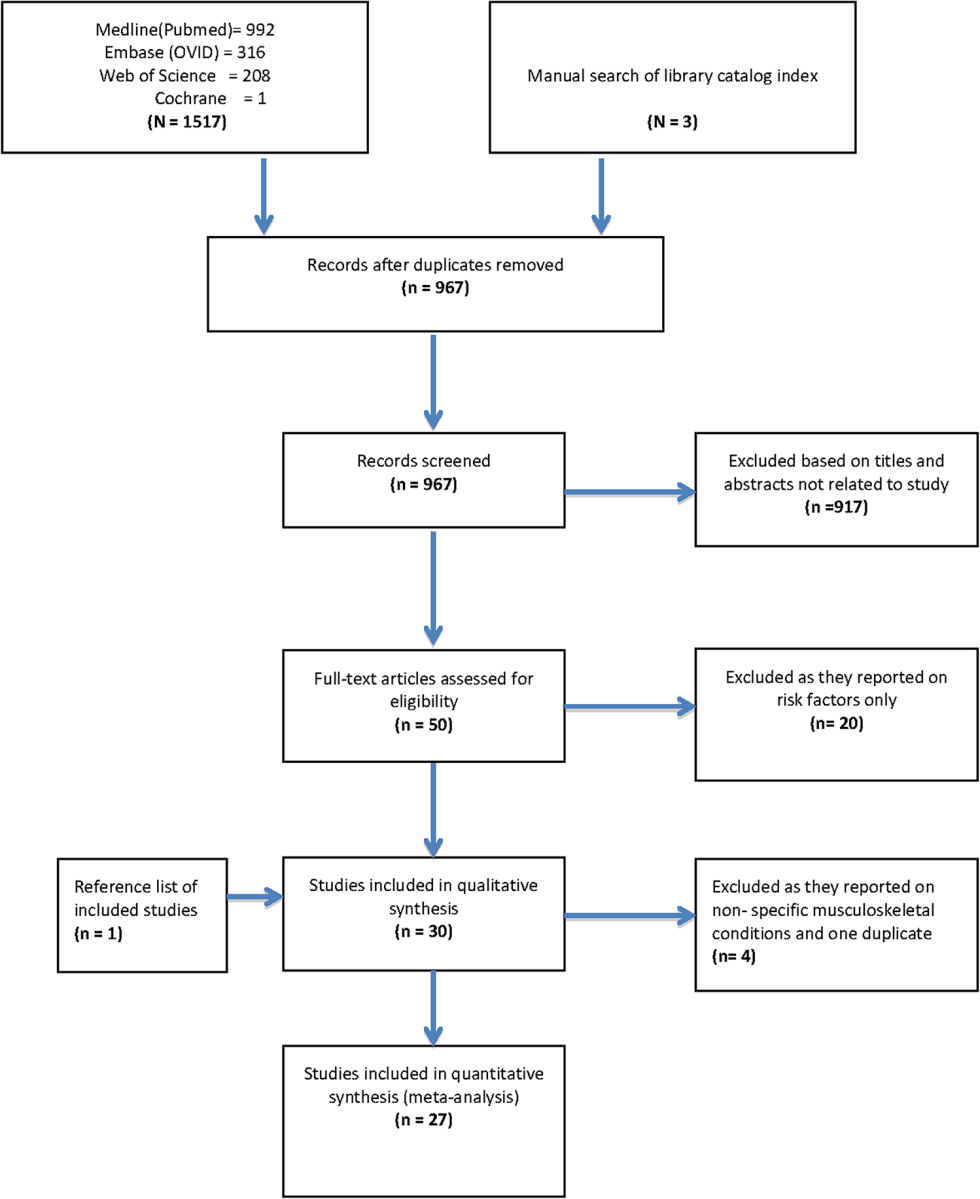
Flow chart showing the search results for cross-sectional studies on the prevalence of arthritis in Africa from 1975 to July 2014.

The flowchart of search results is displayed in Fig 1. Electronic search of four databases yielded 1,517 studies. After removing duplicates, 967 studies remained. We excluded 917 studies by screening titles and abstracts, and retrieved the full texts of 50 remaining studies. We excluded twenty of these studies because they either only reported on risk factors of arthritis or were not prevalence studies or for other reasons (S1 Table) and we remained with 30 studies which reported on arthritis prevalence. At data extraction stage, we excluded four more studies because they reported on non-specific musculoskeletal conditions. We added one more eligible study from reference lists, thus totaling the 27 studies included in this review.

### Characteristics of included studies

**Fig 2:**
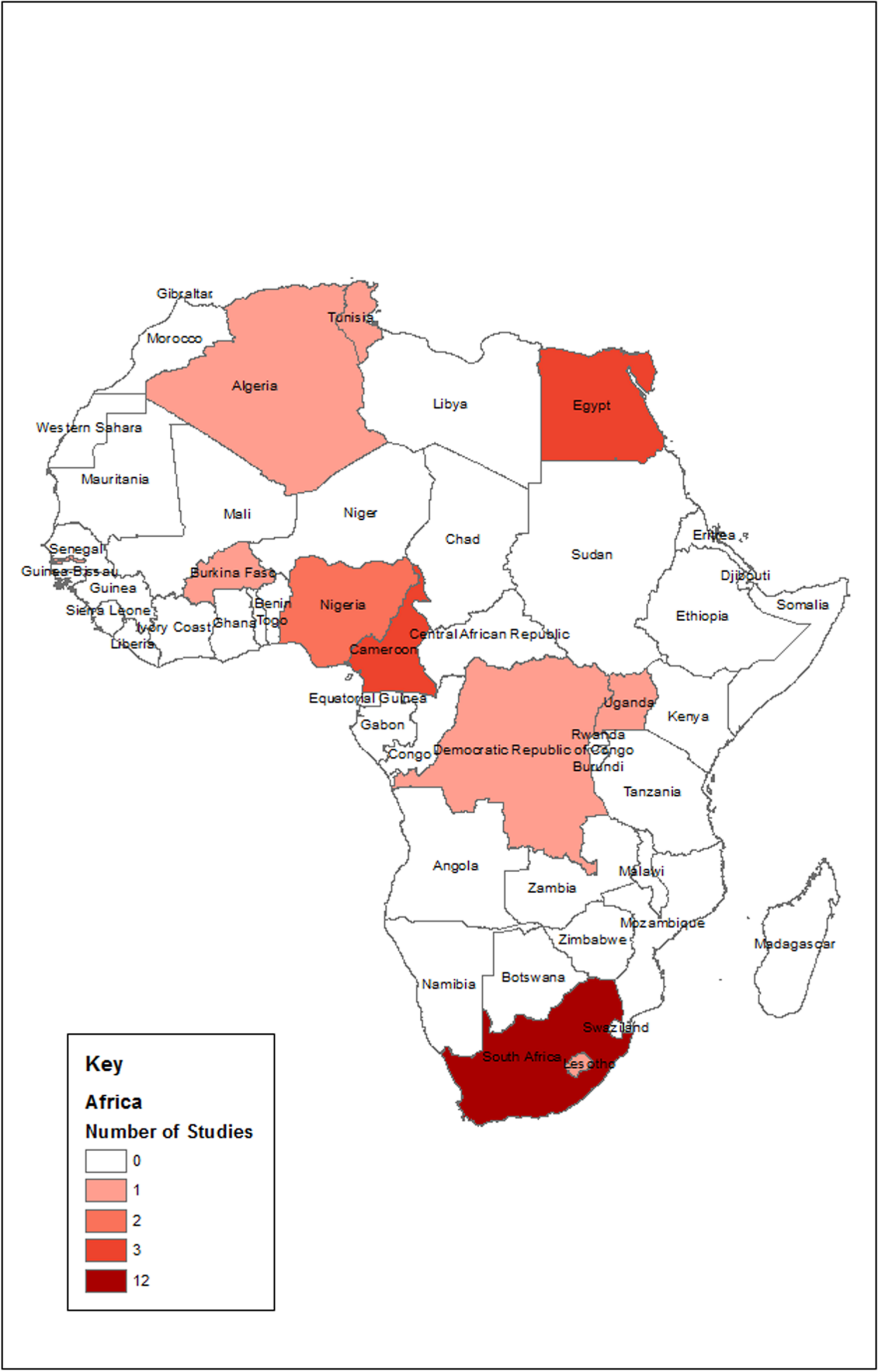
Map of Africa showing the distribution of the number of arthritis prevalence studies from each country published from 1975 to July 2014. Important characteristics of these studies such as country, year of publication, setting, number of participants, age range, type of arthritis and its case definition/diagnosis are presented in Table 3.

The geographical distribution of the number of prevalence studies across the African continent is shown in Fig 2. We included 20 population-based and seven hospital-based cross-sectional studies from 11 countries in Africa, across five geographical regions. Thirteen studies were from the Southern Africa region: twelve from South Africa [18–29] and one from Lesotho [30]. Five studies were from the North African region: three were conducted in Egypt [31–33], one in Tunisia [34] and one in Algeria [35]. Four studies were from West Africa: two from Nigeria [36, 37], one in Burkina Faso [38], and one in Gambia [39]. There was one study from Uganda [40] in East Africa, and four studies from Central Africa: three from Cameroon [41–43] and one from Democratic Republic of Congo (DRC) [44].

**Table 3:** Characteristics of arthritis prevalence studies from African countries published from January 1975 to July 2014.

Study ID	Type of arthritis	Country	Settings	No of participants	Age in years	Case definition/diagnosis
Meyers 1982 [23]	RA, OA	South Africa	Rural, Urban	162	>65	New York criteria
Solomon 1975 Part I [24]	RA; AS	South Africa	Rural, urban	1352	All ages	Modified ROME criteria
Solomon 1975 [26]	RA, PA	South Africa	Urban	964	All ages	Modified ROME criteria
Slimani 2014 [35]	RA	Algeria	Urban	125,253	All ages	1987 ACR
Malemba 2012 [44]	RA	Congo DRC	Urban	5,000	All ages	ACR 1987 Criteria
Beighton 1975 [18]	RA	South Africa	Rural	1183	>15	Modified ROME criteria
Brighton 1988 [21]	RA	South Africa	Rural	543	>18	Modified ROME criteria
Meyers 1977 [22]	RA	South Africa	Rural	577	All ages	Modified ROME criteria
Moolenburgh 1986 [30]	RA	Lesotho	Rural	1070	>15	New York criteria, Modified ROME and ARA criteria
Abdel-Nasser 2004 [32]	RA	Egypt	Rural	5120	>/ = 15	ACR 1987
Silman 1993 [37]	RA, OA	Nigeria	Rural	1994	All ages	ARA 1987
Kaddu-Mukasa 2011 [40]	RA;OA; PA	Uganda	Urban	487	> 18	Not stated. Clinical evaluation by specialist
Singwe-Ngandeu 2007 [41]	RA; OA; Gout; AS;PA;JIA	Cameroon	Urban	536	>/ = 16	International disease classification
Brighton 1985 [20]	OA	South Africa	Rural	543	>18	Modified Kellgreen and Lawrence
Solomon 1976 [27]	OA	South Africa	Rural	293	>35	Kellgreen and Lawrence grade 0–4
Zedini 2014 [34]	OA; knee OA	Tunisia	Urban	2198	> = 65	International Classification of Primary Care (ICPC)
Solomon 1975 Part II [24]	OA	South Africa	Rural	1352	>35	Kellgreen& Lawrence grade 0–4
Ouedraogo 2010 [38]	OA; Gout	Burkina Faso	Urban	366	>18	Not stated
Bija 2014 [42]	Knee OA	Cameroon	Urban	1496	All	1986 ACR
Solomon 1986 [28]	OA	South Africa	Rural	1656	All ages	ROME criteria
Ali Gombe 1996 [36]	OA	Nigeria	Rural	63	60–75	Radiologic diagnosis
Yach 1985 [29]	MJD	South Africa	Rural	2745	All ages	Not stated
Brown 1997 [39]	AS	Gambia	Not stated	1115	>25	ESSG criteria
Abou El Soud 2013 [31]	JIA	Egypt	Rural, urban	3844718	<15	2004 revised ILAR classification
Tayel 1999 [33]	JCA	Egypt	Urban	1500	15-Oct	EULAR criteria
Singwe-Ngandeu 2013 [43]	JIA	Cameroon	Urban	34,782	Children (mean age 10 yrs)	Not reported
Beighton 1977 [19]	Gout	South Africa	Rural, urban	1784	All ages	New York criteria

Seventeen of the studies were published from 1975 to 2000. The remaining ten were published between 2001 until July 2014. All types of arthritis considered in this review were reported by at least one study. A total of 13 studies reported on rheumatoid arthritis [18, 21–24, 26, 30, 32, 35, 37, 40, 41, 44], 12 studies reported on osteoarthritis [20, 23, 25, 27, 28, 34, 36–38, 40–42], 3 studies on ankylosing spondylitis [24, 39, 41], two studies on juvenile idiopathic arthritis [31, 41] and one on juvenile chronic arthritis [33], three studies on psoriatic arthritis [26, 40, 41], two studies on gout [38, 41], and two studies on a South African endemic osteoarthritis known as Mseleni Joint Disease: this is a type of osteoarthritis affecting large joints in mid childhood and affects hundreds of people in Northern KwaZulu-Natal Province of South Africa [28, 29].

Ten studies included participants of all ages, while others included those older than 16 years, with the exception of juvenile arthritis that focused on those younger than 15 years. Two studies include older adults aged 65 years and older [23, 34]. Twelve of the included studies were conducted in rural areas, ten in urban areas and four in mixed rural/urban settings. One study did not specify the setting [31].

### Risk of bias

We assessed each study in ten different domains using the risk of bias tool [14] and the results are shown in Table 4. Of the 27 included studies, our summary assessment was low risk of bias for nine studies (33.3%) [20, 21, 30–33, 35, 41, 42], moderate risk of bias for 11 studies (40.8%) [18, 19, 22–28, 36, 44] and high risk of bias for seven studies (25.9%) [23, 29, 34, 37–40, 43]. We also found that 81.5% (22/27) of the studies did not represent the national population.

**Table 4:** Risk of bias assessment of included studies using the Hoy 2012 tool [14].

Study ID	Representation	Sampling	Random selection	Non-response bias	Data collection	Case definition	Reliability of tool	Method of data collection	Prevalence period	Numerators and denominators	Summary assessment
Zedini 2014 [34]	high	unclear	low	Unclear	low	unclear	unclear	low	low	low	high
Bija 2014 [42]	low	unclear	unclear	Low	low	low	low	low	low	low	low
Slimani 2014 [35]	low	low	low	Low	low	low	low	low	low	unclear	low
Singwe-Ngandeu 2013[43]	high	low	low	Unclear	high	unclear	low	low	unclear	high	high
Abou El Soud 2013 [31]	low	low	high	Unclear	low	low	low	low	low	low	low
Malemba 2012 [44]	high	low	low	Unclear	low	low	low	low	unclear	low	moderate
Kaddu-Mukasa 2011 [40]	high	low	high	Unclear	low	unclear	unclear	low	unclear	low	high
Ouedraogo 2010 [38]	high	high	high	Unclear	low	unclear	unclear	unclear	unclear	unclear	high
Singwe-Ngandeu 2007 [41]	high	low	high	Low	low	low	low	low	unclear	low	low
Abdel-Nasser 2004 [32]	high	low	low	Low	low	low	low	low	low	low	low
Tayel 1999 [33]	unclear	low	low	Low	low	low	low	low	unclear	low	low
Brown 1997 [39]	high	unclear	high	Unclear	low	low	low	high	unclear	low	high
Ali Gombe 1996 [36]	high	low	high	Low	low	low	low	low	unclear	unclear	moderate
Silman 1993 [37]	high	low	low	High	low	low	high	high	unclear	high	high
Brighton 1988 [21]	high	low	low	Low	low	low	low	low	unclear	low	low
Moolenburgh 1986 [30]	low	low	low	High	low	low	low	low	unclear	low	low
Solomon 1986 [28]	high	unclear	low	Unclear	low	low	low	low	unclear	low	moderate
Brighton 1985 [20]	high	low	low	Low	low	low	low	low	unclear	low	low
Yach 1985 [29]	high	low	low	Unclear	high	high	unclear	high	unclear	low	high
Meyers 1982 [23]	high	low	low	Low	low	low	low	low	unclear	high	moderate
Beighton 1977 [19]	low	low	low	Unclear	low	low	low	low	unclear	unclear	moderate
Meyers 1977 [22]	high	low	low	Low	low	low	low	low	unclear	high	moderate
Solomon 1976 [27]	high	low	low	Unclear	low	low	low	low	unclear	unclear	moderate
Beighton 1975 [18]	high	low	low	Unclear	low	low	low	low	unclear	low	moderate
Solomon 1975 Part I [24]	high	low	low	Unclear	low	low	low	low	unclear	unclear	moderate
Solomon 1975 Part II [25]	high	low	low	Unclear	low	low	low	low	unclear	unclear	moderate
Solomon 1975 [26]	high	low	low	High	low	low	low	low	unclear	low	moderate

### Prevalence results

#### Rheumatoid arthritis–Population-based studies

Out of the 13 studies reporting rheumatoid arthritis prevalence, 11 were population-based studies. Five of these eleven population-based studies were done in urban settings [23, 24, 26, 35, 44] and were pooled in a meta-analysis that yielded significant heterogeneity (I^2^ = 86.3%, p<0.001).

**Fig 3:**
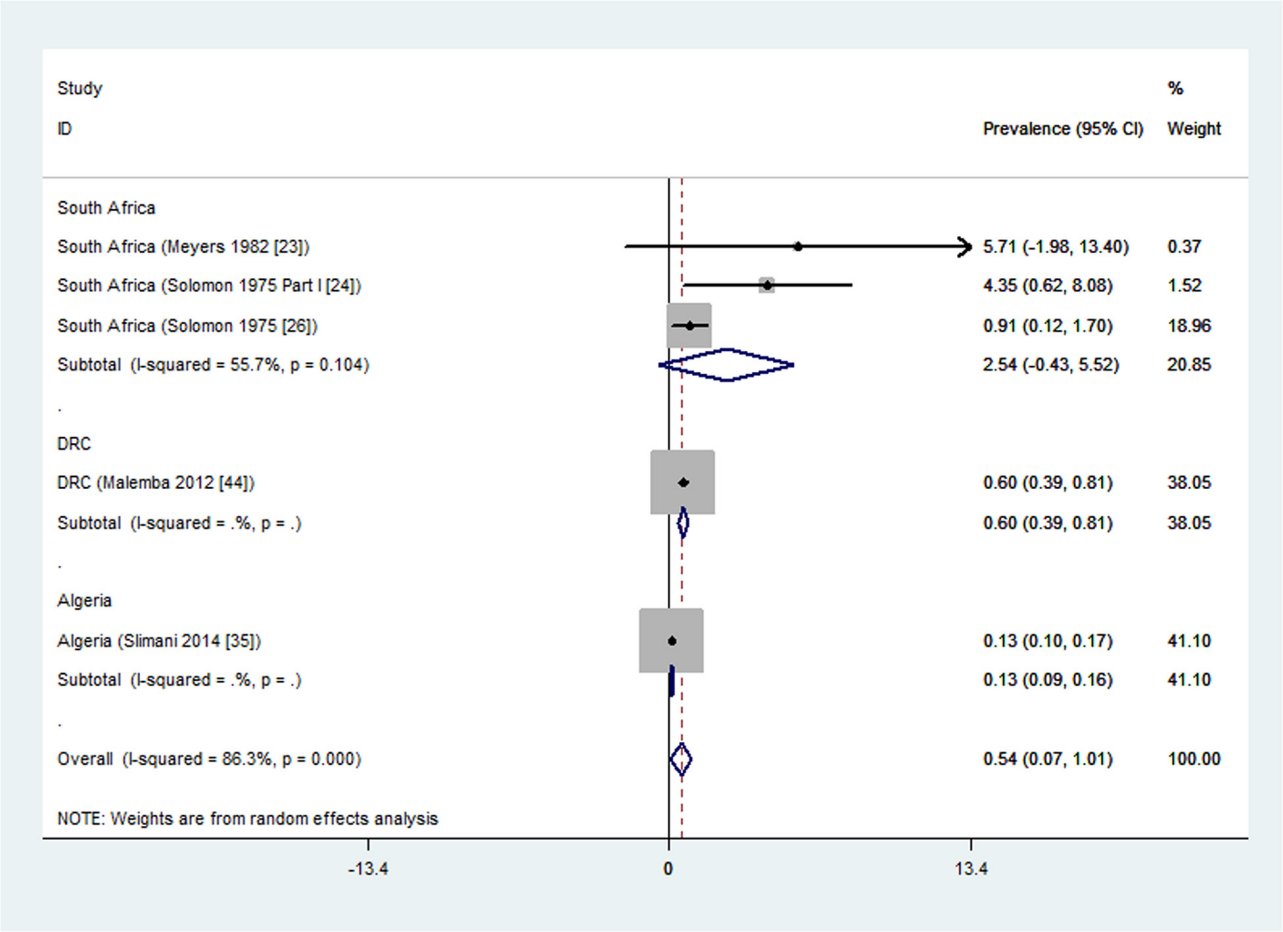
Forest plot showing the meta-analysis of urban population-based prevalence (%) of rheumatoid arthritis in African countries.

We performed subgroup analysis with respect to country and there was no more significant heterogeneity within countries and we therefore report results per country (Fig 3). The prevalence ranged from a minimum of 0.13% (95% CI 0.10 to 0.17) in Algeria [35], 0.6%(95% CI 0.40 to 0.80) in the Democratic Republic of Congo [44], to a maximum meta-analysis result of 2.54% (95% CI -0.43 to 5.52) in South Africa [23, 24, 26]. The overall rheumatoid arthritis prevalence for South Africa was a meta-analysis of three studies with individual prevalence estimates ranging from 0.91% [26], 4.35% [24], to 5.71% [23]; with moderate but statistically non-significant heterogeneity (I^2^ = 55.7%, p = 0.104). All these three South African studies that were meta-analyzed had moderate risk of bias assessment. However, one South African study reporting the highest prevalence (5.71%) only included adults aged over 65 years [23].

Eight studies, all population-based, reported rheumatoid arthritis prevalence in rural settings. One study from Nigeria [37] reported no cases (prevalence of zero) and was excluded from meta-analysis. The prevalence estimates from the seven remaining population-based studies were pooled in a meta-analysis that yielded significant statistical heterogeneity (I^2^ = 74.9%, p = 0.001).

**Fig 4:**
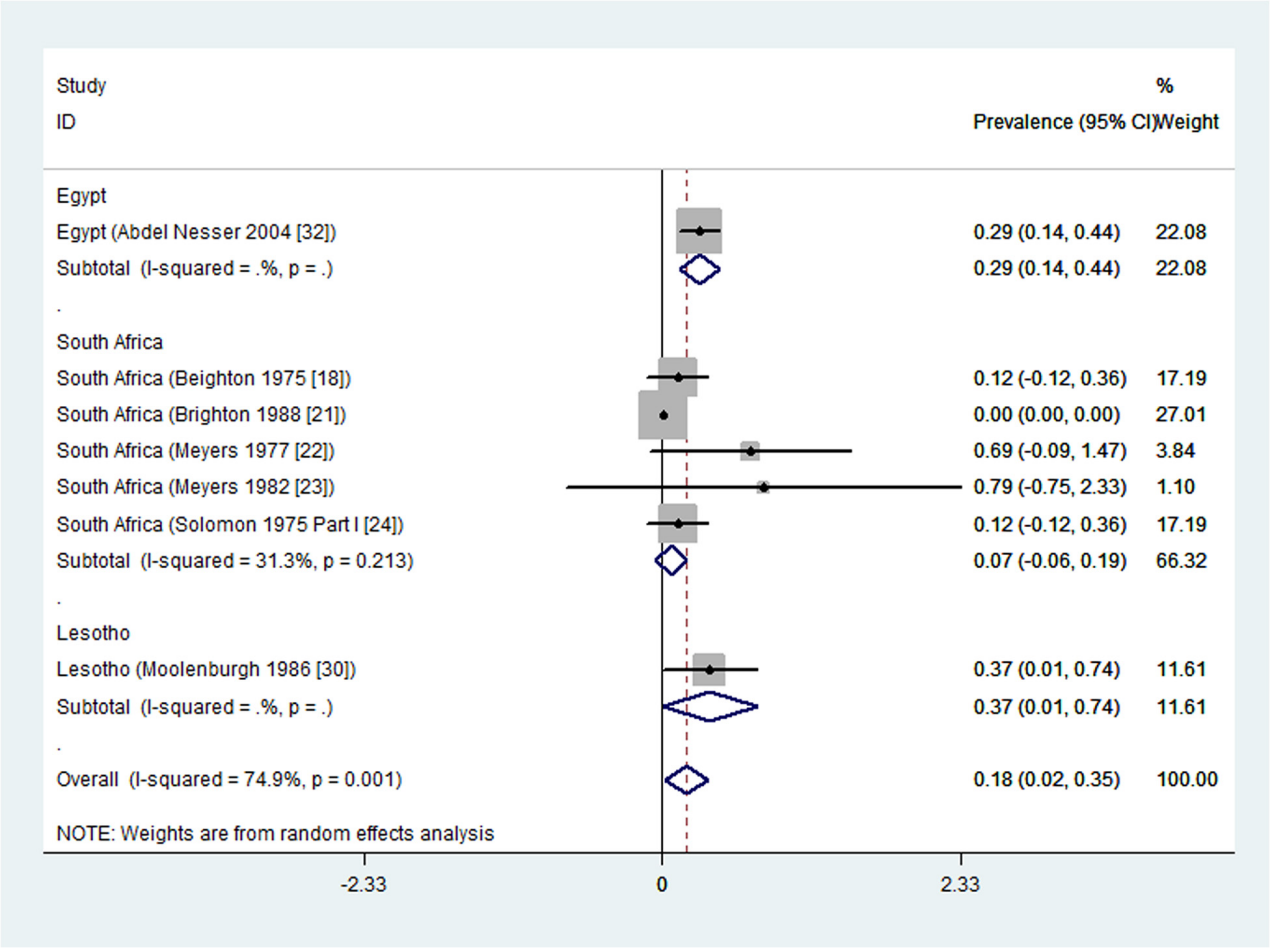
Forest plot showing the meta-analysis of prevalence (%) of rheumatoid arthritis from rural population-based studies in African countries.

A subgroup analysis with respect to country resulted in statistically non-significant heterogeneity within countries (Fig 4). The rheumatoid arthritis prevalence in rural settings per country ranged from a minimum meta-analysis overall prevalence of 0.07% (95% CI -0.06 to 0.19) in South Africa [18, 21–24], 0.29% (95% CI 0.14 to 0.44) in Egypt [32], to a maximum of 0.37% (95% CI 0.01 to 0.74) in Lesotho [30]. The five South African studies pooled in the meta-analysis had low (one study) to moderate (four studies) risk of bias.

Significant statistical heterogeneity in the overall meta-analysis is probably explained by the differences amongst regions or countries.

#### Rheumatoid arthritis–Hospital-based studies

Two hospital-based studies, both from urban settings, reported on rheumatoid arthritis prevalence [40, 41]. One study found a prevalence of 0.67% (95% CI -0.25 to 1.59) among HIV-infected adults attending a clinic in urban Uganda [40] and the other one found a prevalence of 0.1% (95% CI 0.05 to 0.16) among HIV-infected adults attending an infectious disease clinic at Mulago hospital in Cameroon [41]. These two studies were not pooled in a meta-analysis because while the Ugandan study had high risk of bias, the Cameroon study had a low risk of bias.

#### Osteoarthritis–Population-based studies

Four population-based studies reported on the prevalence of osteoarthritis from a rural setting, without specifying the type of joint [20, 23, 27, 37]. A meta-analysis of these studies resulted in significant statistical heterogeneity and their results are therefore reported separately (Table 5). There were three studies from South Africa; two with similar osteoarthritis prevalence estimates of 29.5% (95% CI 25.64 to 33.30) [20] and 29.7% (95% CI 24.46 to 34.92) [27] and one with a higher prevalence of 82.7% (95% CI 76.10 to 89.26) [23] among South African adults older than 65 years.

**Table 5:** Osteoarthritis prevalence (%) and corresponding 95% confidence intervals for urban and rural African settings from population-based studies.

Reference	Country	Rural	Urban	Total
Brighton 1985 [20]	South Africa	29.5 (25.64–33.30)		
Solomon 1976 [27]	South Africa	29.7 (24.46–34.92)		
Meyers 1982 [23]	South Africa	82.7 (76.10–89.26)	55.1 (40.74–73.54)	77.2 (70.70–83.62)
Silman 1993 [37]	Nigeria	0.4 (0.12–0.68)		
Kaddu-Mukasa 2011 [40]	Uganda	0.3 (-0.32–0.98)		
Bija 2014 [42]	Tunisia		14.8 (13.31 to 16.27)	

There was also one population-based study from Nigeria reporting an osteoarthritis prevalence of 0.4% (95% CI 0.12 to 0.68) [37].

Only one population-based study reported on osteoarthritis prevalence of 55.1% (95% CI 40.74 to 73.54) among adults aged over 65 years in an urban setting in South Africa [23].

#### Osteoarthritis–Hospital-based studies

Two hospital-based studies reported on osteoarthritis prevalence, without specifying the type of joint [34, 40]. Both of them were from urban settings. One study from Uganda [40] found a prevalence of 0.3% (95% CI -0.32 to 0.98) among HIV infected adults attending an infectious disease clinic. The other study reported a prevalence of 14.8% (95%CI: 13.31 to 16.27) from an elderly population of urban dwellers in Tunisia [34]. These two hospital-based studies on osteoarthritis prevalence could not be combined in meta-analysis due to significant statistical heterogeneity.

#### Osteoarthritis of the knee–Population-based studies

There was one population-based study from South African rural setting reporting a knee osteoarthritis prevalence of 33.1% (95% CI 27.70 to 38.50) among adults aged over 35 years [25].

#### Osteoarthritis of the knee–Hospital-based studies

There were three urban hospital-based studies reporting on osteoarthritis of the knee. One study from Burkina Faso [38] reported a knee osteoarthritis prevalence of 0.5% (95% CI -0.20 to 1.20) among HIV-infected adult patients undergoing highly active antiretroviral therapy. The second study [34] reported a prevalence of 4.7% (95%CI 3.84 to 5.62) among the elderly in primary care in Tunisia and the third study [42] reported a prevalence of 9.9% [95%CI 8.38 to 11.40] among hospital patients with musculoskeletal conditions in Cameroon. We did not pool these studies in a meta-analysis due to significant statistical heterogeneity.

#### Osteoarthritis of the hip–Population-based studies

**Fig 5:**
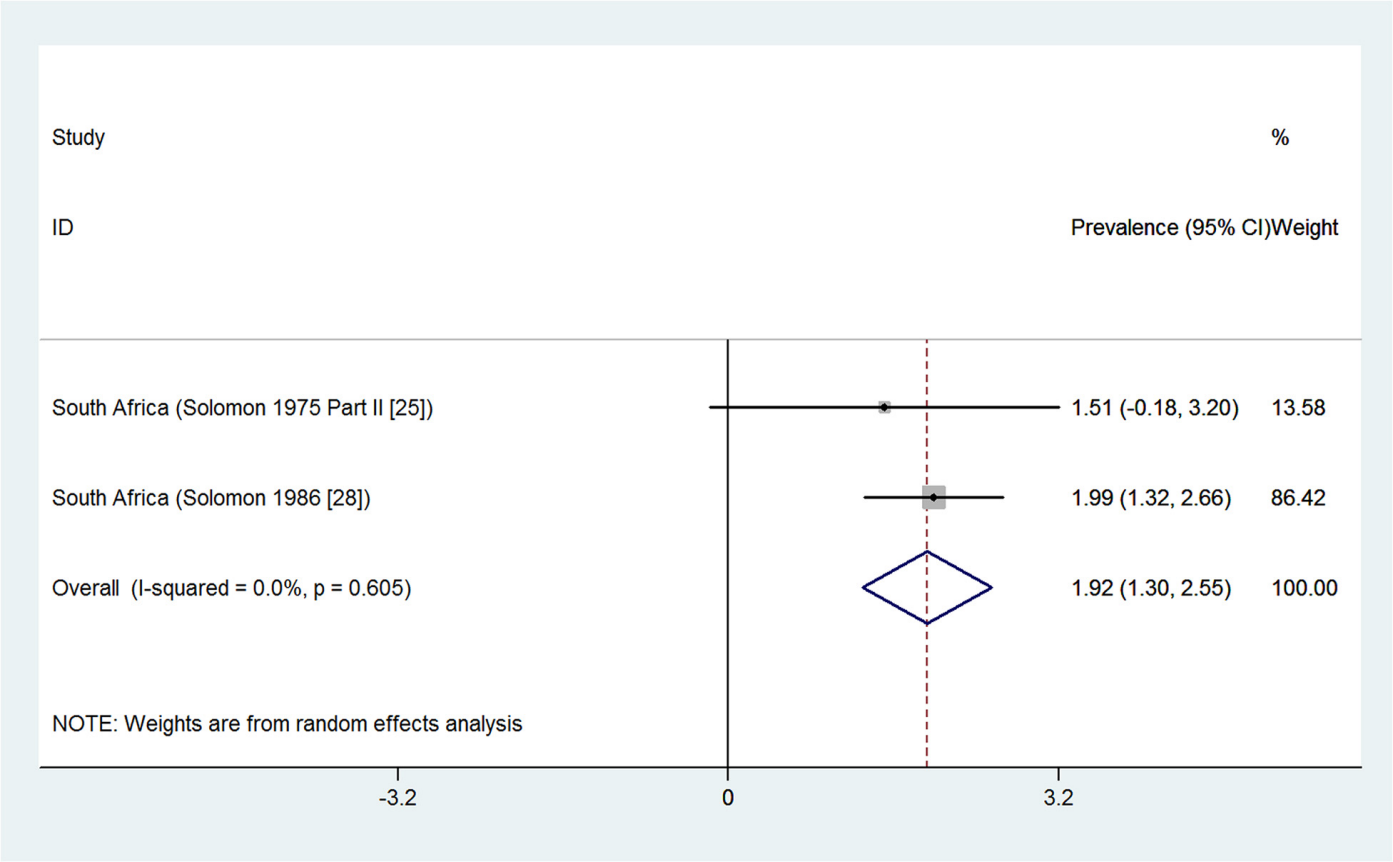
Forest plot showing the meta-analysis of population-based prevalence (%) of osteoarthritis of the hip in rural settings of South Africa.

Two population-based studies from South Africa reported on the prevalence of osteoarthritis of the hip from rural settings [25, 28] (Fig 5). The two studies were pooled in a meta-analysis that found an overall prevalence of 1.9% (95%CI 1.30% to 2.55%) and there was no significant statistical heterogeneity detected between the studies. The two studies included adults aged over 55 years [25] and 60 years [28]. Both studies had moderate risk of bias assessment.

#### Osteoarthritis of the hip–Hospital-based studies

One hospital-based study reported a prevalence of osteoarthritis of the hip of 4.8% (95%CI -0.50 to 10.10) among 63 male patients aged 60 to 75 years who had undergone urography for non-arthritic conditions at an urban teaching hospital in Nigeria [36].

#### Osteoarthritis of the DIP, MCP, PIP, first MTP and limbs–Population-based studies

Among adults aged 35 years and above in a rural setting in South Africa, a study found prevalence of osteoarthritis of the Distal interphalangeal (DIP) of 38% (95% CI 32.50 to 43.50), Metacapophalangeal (MCP) of 14.7% (95% CI 10.70 to 18.70), Proximal interphalangeal (PIP) of 14% (95% CI 10.10 to 17.90), first Metatarsophalangeal (MTP) joints of 21.3% (95% CI 16.70 to 25.90), and limbs of 0.9% (95% CI 0.70 to 1.10) [25].

#### Endemic Osteoarthritis (Mseleni Joint Disease)–Population-based studies

One population-based study from a rural setting in South Africa found a prevalence of 5.9% (95% CI 5.10 to 6.70) for an endemic osteoarthritis known as Mseleni Joint Disease [29].

#### Ankylosing Spondylitis–Population-based studies

Two population-based studies reported on the prevalence of ankylosing spondylitis. One study from rural South Africa reported a prevalence of 0.10% (95% CI -0.10 to 0.30) [24]. The second study reported no cases of ankylosing spondylitis in the Fula ethnic group in the Gambia [39].

#### Ankylosing Spondylitis–Hospital-based studies

One hospital-based study also reported a prevalence of ankylosing spondylitis of 0.03% (95%CI: 0.00 to 0.06) among outpatients of a rheumatology clinic at the urban Yaounde General Hospital, Cameroon [41].

#### Juvenile arthritis–Population-based studies

Two population-based studies reported on the prevalence of juvenile arthritis in Egypt. The first study reported a prevalence of juvenile idiopathic arthritis of 0.00343%(95%CI 0.0031 to 0.0043) among children in Sharkia Governorate, Egypt [31]. The second study from an urban setting found a prevalence of 0.33% (95% CI 0.04 to 0.62) among school children aged 10 to 15 years enrolled in primary and preparatory schools in Alexandria, Egypt [33]. The two studies were not meta-analyzed due to significant statistical heterogeneity.

#### Juvenile arthritis–Hospital-based studies

Two hospital-based studies from Cameroon reported the prevalence of juvenile idiopathic arthritis. The first study reported a prevalence of 0.01% (95%CI -0.01 to 0.02) among outpatients of a rheumatology clinic at the urban Yaounde General Hospital, Cameroon [41]. The second study reported a prevalence of 0.1% (95%CI 0.07 to 0.13] among urban Cameroonian children and adolescents suffering from rheumatic disorders [43]. These two studies were not pooled in a meta-analysis due to significant statistical heterogeneity.

#### Psoriatic arthritis–Population-based studies

One population-based study from urban South Africa found a prevalence of psoriatic arthritis of 4.4% (95% CI 2.70 to 6.10) [26].

#### Psoriatic arthritis–Hospital-based studies

A hospital-based study found a prevalence of 1% (95% CI -0.10 to 2.10) among HIV-infected adult patients attending a clinic in urban Uganda [40]. The second study reported a prevalence of 0.01% (95%CI -0.01 to 0.02) among outpatients of a rheumatology clinic at the urban Yaounde General Hospital, Cameroon [41]. The two studies were not pooled in a meta-analysis due to significant statistical heterogeneity.

#### Gout–Population-based studies

In South Africa, a population-based study found a prevalence of 0.70% (95%CI 0.00% to 1.40%) among Caucasians. No cases of gout were found among black Africans [19].

#### Gout–Hospital-based studies

In Burkina Faso, a hospital-based study found a gout prevalence of 0.30% (95%CI -0.30% to 0.90%) among HIV-infected patients undergoing antiretroviral therapy [38].

## Discussion

We carried out this systematic review with the objective of assessing the prevalence of six types of arthritis in Africa. We found 27 studies from 11 countries in Africa using a detailed search of electronic databases and manual searches. However, these studies were unevenly distributed (12 from South Africa), and approximately half of them (13/27, 48%) were published before 2000. Fourteen of the included studies (14/27, 52%) were published from 2001 to date, emphasizing the paucity of data on arthritis prevalence in Africa.

The wide range of prevalence found could have been due to the diverse cultural and geographic nature of Africa, and the methodological differences between studies. The majority of the included studies (20/27, 74%) were population-based studies and the remaining seven studies were hospital-based. Population-based and hospital-based studies were reported separately. We did double data screening and extraction and performed rigorous meta-analysis with investigation of heterogeneity through subgroup analysis.

We summarize the key findings of this study from the population-based studies. The prevalence estimates of rheumatoid arthritis in urban settings, per country, varied from 0.13% in Algeria [35], 0.6% in the Democratic Republic of Congo [44] to a maximum meta-analysis overall prevalence of 2.5% in South Africa (meta-analysis of 3 studies [23, 24, 26]). The overall prevalence could not be reported because of significant heterogeneity between the studies. In rural settings, the rheumatoid arthritis prevalence per country ranged from zero in Nigeria [37], 0.07% in South Africa (a meta-analysis of five studies [18, 21–24]), 0.29% in Egypt [32], to a maximum of 0.37% in Lesotho [30]. These prevalence estimates are in the same range as the previously reported overall estimate of 0.36% for rheumatoid arthritis in Africa [45]. The prevalence estimates of RA found in this systematic review are comparable with those outside Africa: a systematic review [46] found median prevalence estimate for the total population in South Europe of 0.33% (range 0.31 to 0.50), for North European countries 0.50% (range 0.44 to 0.80), for developing countries outside Africa 0.35% (range 0.24 to 0.36), a study in North America found a prevalence of 0.11%.

In rural settings, osteoarthritis prevalence ranged from 0.4% in Nigeria [37], 29.5% [20] and 29.7% [27] (both in South Africa), to a maximum of 82.7% [23] in a South African study involving adults aged over 65 years. This same study reported a prevalence of 55.1% for osteoarthritis in an urban setting among the older adults aged above 65 years [23]. A meta-analysis of two population-based studies from South Africa of adults over 55 years gave a combined prevalence of 1.9% for osteoarthritis of the hip [25, 28]. Other results include highest prevalence estimates of 33.1% for knee osteoarthritis in South Africa [25], 0.10% for ankylosing spondylitis in South Africa [24], 4.40% for psoriatic arthritis in South Africa [26], 0.70% for gout in South Africa [19] and 0.33% for juvenile idiopathic arthritis in Egypt [33]. A systematic review [47] found a median prevalence of psoriatic arthritis of 0.0018% (range 0.00001 to 0.0042) for studies outside Africa. Another systematic review [48] found the prevalence of juvenile idiopathic arthritis ranging from 0.000038% to 0.004% for studies outside Africa.

The main limitation of this study is the paucity of reliable prevalence data for different types of arthritis in Africa. The risk of bias assessment results showed that most of the included studies were not representative of the national population and this limits the generalizability of prevalence estimates obtained from this study. In addition, the available prevalence data is heterogeneous thereby making the meta-analysis invalid. Potential sources of heterogeneity include different diagnostic criteria, regional differences, rural versus urban settings, and the different age groups. Although a subgroup analysis based on diagnostic criteria was not done, direct comparability of results may be misleading due to differences in case definition. The use of standardized criteria as defined by WHO in 2003 [49] is warranted for future studies to allow for comparability across region and gender. Similarly the zero prevalence reported for juvenile idiopathic arthritis and other rare arthritis may not necessarily mean the absence of the disease, but highlight the rarity of the disease, requiring larger sample sizes. This may have resulted in under-estimation of arthritis prevalence in Africa.

Due to the sparse available data and the non- national representation exhibited by the majority of the included studies, the findings of this review may not be generalized to the whole African continent. There was good agreement between authors regarding the risk of bias assessments of included studies using the Hoy 2012 tool. However, the tool did not provide an objective means of assessing the overall bias. For this study we decided to allocate each of the ten parameters in the risk of bias tool an equal weight. Thus the overall assessment of bias was dependent on the number of high risk parameters out of the ten parameters. Most of the authors could not be reached due to the less effective means of communication given in the era in which those studies were done.

With Africa’s attention focused on infectious diseases and maternal and child health, the burden of non-communicable diseases has gradually increased. Africa is now at risk of a double burden of communicable and non-communicable disease, with the latter estimated to cause more than 60% mortality by 2030 [10]. Further population-based studies on arthritis prevalence in Africa are therefore needed.

## Conclusions

### Implications for research

This systematic review has confirmed the lack of prevalence data on arthritis in Africa, and has inadvertently exposed the question of reliability of the available data. Available reports on arthritis are too old to reflect present trends of the disease. The African League of Associations for Rheumatology is encouraged to lead a solution to the need of a Standard Demographic Health Survey in the five regions of Africa, using standardized diagnostic criteria, where applicable, which would help to fill these gaps and address the true burden of arthritis in Africa.

### Implications for practice

The presentation of arthritis is painful swelling at the joints for which most patients are placed on analgesic under the broad diagnosis of musculoskeletal condition. Further investigation and definitive diagnosis of musculoskeletal could avoid under-reporting of arthritis, especially now that affordable drugs are available [50]. Thus a standardized, easy to apply, diagnostic criteria for case definitions is paramount, not just for rheumatologists, but also for physicians practicing at the primary healthcare facilities in the rural areas where the vast majority of the African population still resides.

### Implications for policy makers

With the attention of policy makers directed towards emerging and re-emerging infectious diseases in Africa, non-communicable diseases are on the increase. The burden of arthritis and its implication on productivity over time need to be assessed.

## Supporting Information

S1 Table Excluded studies with reasons for exclusion from the systematic review on the prevalence of arthritis in Africa. (DOCX)

S2 Table PRISMA checklist for the systematic review on the prevalence of arthritis in Africa. (DOC)
