# Supplementary material for: Prevalence of Arthritis in Africa: A Systematic Review and Meta-Analysis
Source: PLoS One. 2015 Aug 4;10(8):e0133858. doi: 10.1371/journal.pone.0133858 (PMC4524637; doi:10.1371/journal.pone.0133858)
Supplement: S1 Table — (DOCX) [file pone.0133858.s001.docx]

**S1 Table. Excluded studies with reasons for exclusion from the systematic review on the prevalence of arthritis in Africa**

| **Study citation** | **Reason for exclusion** |
| --- | --- |
| 1.    Agarwal, P., et al. (2013). "Depression treatment patterns among individuals with osteoarthritis: a cross sectional study." BMC Psychiatry **13**(1): 121. | Not a prevalence study |
| 2.    Parker, S. J., et al. (2011). "A comparison of the arthritis foundation self-help program across three race/ethnicity groups." Ethn Dis **21**(4): 444-450. | Not an African study |
| 3.    Powell, A. M., et al. (2005). "Discoid lupus erythematosus with secondary amyloidosis." Br J Dermatol **153**(4): 746-749. | Not a prevalence study |
| 4.    Salah, S., et al. (2009). "Juvenile idiophatic arthritis, the Egyptian experience." Journal of Medical Sciences **9 (2)**: 98-102. | Not a prevalence study |
| 5.    Kalichman, L., et al. (2010). "Radiographic hand osteoarthritis in two ethnic groups living in the same geographic area." Rheumatol Int **30**(11): 1533-1536. | Not an African study |
| 6.    Mijiyawa, M. (1995). "Gout in patients attending the rheumatology unit of Lome Hospital." Br J Rheumatol **34**(9): 843-846. | Not a prevalence study |
| 7.    Ekwom, P. E., et al. (2010). "Prevalence and characteristics of articula manifestations in human immunodeficiency virus infection." East African medical journal **87 (10)**: 408-414. | Study on non-specific HIV athropathy |
| 8.    Njobvu, P. and P. McGill (2005). "Human immunodeficiency virus related reactive arthritis in Zambia." J Rheumatol **32**(7): 1299-1304. | Study on non-specific Spondyloarthropathies |
| 9.    Adebajo, A. O. (1995). "Osteoarthritis." Baillieres Clin Rheumatol **9**(1): 65-74. | A review article |
| 10.    Davis, P. and M. Stein (1991). "Human immunodeficiency virus-related connective tissue diseases: a Zimbabwean perspective." Rheum Dis Clin North Am **17**(1): 89-97. | Not a prevalence study - only risk factors |
| 11.    El Garf, A., et al. (2012). "Prevalence and clinical presentations of hepatitis C virus among patients admitted to the rheumatology ward." Rheumatol Int **32**(9): 2691-2695. | Not a prevalence study - only risk factors |
| 12.    Essackjee, K., et al. (2013). "Prevalence of and risk factors for chronic arthralgia and rheumatoid-like polyarthritis more than 2 years after infection with chikungunya virus." Postgrad Med J **89**(1054): 440-447. | Not a prevalence study - only risk factors |
| 13.    Feki, S., et al. (2012). "[Prevalence and diagnostic value of antinuclear antibodies without identified antigenic target: A retrospective study of 90 patients.]." Rev Med Interne **33**(9): 475-481. | Not a prevalence study - only risk factors |
| 14.    Frikha, F., et al. (2009). "Destructive arthritis in Behcet's disease: a report of eight cases and literature review." Int J Rheum Dis **12**(3): 250-255. | Case report |
| 15.    Li, X. J., et al. (2009). "Risks of Rheumatic Diseases in First- and Second-Generation Immigrants in Sweden." Arthritis and Rheumatism **60**(6): 1588-1596. | Not an African study |
| 16.    Petri, M., et al. (2009). "Prevalence of flare and influence of demographic and serologic factors on flare risk in systemic lupus erythematosus: a prospective study." J Rheumatol **36**(11): 2476-2480. | Not a prevalence study - only risk factors |
| 17.    Abdulraheem, I. S., et al. (2011). "Prevalence and correlates of physical disability and functional limitation among elderly rural population in Nigeria." Journal of Aging Research **2011**(369894). | Study on limitations of non-specific arthritis |
| 18.    Jelsma, J., et al. (2002). "Disability in an urban black community in Zimbabwe." Disabil Rehabil **24**(16): 851-859. | Study on non-specific arthritis |
| 19. Elleuch, M. H., et al. (2008). "Knee osteoarthritis in 50 former top-level soccer players: a comparative study." Ann Readapt Med Phys **51**(3): 174-178. | Not a prevalence study |
| 20. Hashaad, N. I., et al. (2012). "Interleukin-18 promoter polymorphisms in Egyptian patients with rheumatoid arthritis." Egypt J Immunol **19**(2): 13-24. | Not a prevalence study - only risk factors |
